# Management of device embolization following left atrial appendage closure: Two cases and a review of the literature

**DOI:** 10.1002/joa3.70139

**Published:** 2025-08-01

**Authors:** Ahmet Kivrak, Ahmet Hakan Ates, Ugur Canpolat, Mert Dogan, Cem Coteli, Hikmet Yorgun, Mehmet Levent Sahiner, Ergun Barıs Kaya, Kudret Aytemir

**Affiliations:** ^1^ Department of Cardiology Hacettepe University Faculty of Medicine Ankara Turkey

**Keywords:** atrial fibrillation, device embolization, left atrial appendage closure, percutaneous retrieval techniques

## Abstract

**Objectives:**

Device embolization (DE) following left atrial appendage closure (LAAC) is a severe but uncommon complication, and limited data address optimal management strategies for this condition. This review presents two cases of device embolization (DE) following LAAC and discusses risk factors, incidence, and management strategies through a literature‐based approach.

**Methods:**

A comprehensive literature review was conducted, including studies focused on DE after LAAC, examining percutaneous and surgical retrieval techniques, procedural success, and patient outcomes.

**Results and Conclusion:**

DE incidence ranges from 0.6% to 1.5%, with improper device sizing and anatomical factors as primary risk factors. Percutaneous retrieval, through transseptal or transarterial approaches, demonstrates high procedural success rates, while surgical retrieval remains an option for complex cases. Our review suggests that with experienced operators, tailored percutaneous strategies effectively manage DE following LAAC.

## Graphical abstract

Device embolization following left atrial appendage closure occurs in 0.6%–1.5% of cases and represents a rare but serious complication that can be effectively managed through tailored percutaneous retrieval strategies with high procedural success rates when performed by experienced operators.

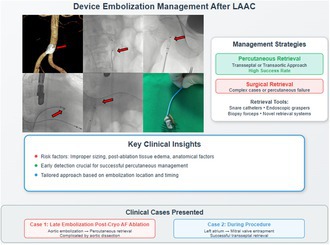

## 1 INTRODUCTION

Atrial fibrillation (AF) is the most common cardiac arrhythmia, affecting millions of people worldwide, and is associated with increased morbidity and mortality.^1^ AF significantly increases the risk of ischemic stroke, accounting for approximately 20%–30% of all strokes.^2^ Although oral anticoagulation (OAC) is the standard treatment to reduce stroke risk in AF patients, many patients are ineligible for or unable to maintain long‐term OAC because of the risk of significant bleeding or other contraindications. The left atrial appendage (LAA) becomes a critical target for stroke prevention in those high‐risk patients. Percutaneous LAA closure (LAAC) has been developed as an alternative to OAC, offering a mechanical and interventional solution to prevent thromboembolic events.^3^ While LAAC has demonstrated efficacy in reducing stroke risk, it is not without its procedural risks. Among these, device embolization (DE) represents one of the most severe and challenging complications, often requiring urgent intervention. Despite this, there remains a significant gap in the literature regarding the optimal management of DE when it occurs and a need for more consensus on best practices for its prevention. Existing studies have focused on procedural outcomes and long‐term efficacy, with fewer addressing the specific techniques and strategies to manage DE effectively.

In this paper, which includes two case reports with DE after LAAC and a literature review, we aim to address the gaps in the prevention and management of DE after LAAC and give details of challenges encountered and strategies employed to manage DE successfully.

## 2 CASES

### 2.1 Case 1

A 63‐year‐old male patient with paroxysmal atrial fibrillation (PAF), cerebral amyloid angiopathy, a history of ischemic stroke, and hypertension was admitted to our clinic with complaints of dyspnea and palpitations. The patient received apixaban at a dose of 2,5 mg twice daily for anticoagulation. On physical examination, no significant abnormalities were found. The patient weighed 74 kg, had a height of 170 cm, and had a body mass index (BMI) of 25.6 kg/m^2^ (Table 1). Laboratory tests showed a serum creatinine level of 0.9 mg/dL and an estimated glomerular filtration rate (eGFR) of over 60 mL/min/1.73 m^2^, indicating normal kidney function. The results of the complete blood count were also within normal limits. Electrocardiography (ECG) revealed normal sinus rhythm with a heart rate of 73 beats per minute. Transthoracic echocardiography (TTE) showed a left ventricular ejection fraction (LVEF) of 60%, mild mitral and tricuspid regurgitation, and a left atrial diameter of 38 mm. The neurology department evaluated the patient, noting a history of cerebral amyloid angiopathy, which placed him at high risk for bleeding with continued OAC therapy. Given that the patient was also symptomatic, we decided to proceed with catheter ablation for AF and LAAC to manage both AF and reduce the risk of future bleeding.

**TABLE 1:** Baseline demographic and clinical characteristics of the patients.

	Age/gender	Landing zone (TEE)	Device, size (mm)	DE time	DE area	Management	Complication
Case‐1	63/M	17 mm	Amulet, 20 mm	1 week	Terminal aorta	Percutaneous transaortic trapping	Descending aorta dissection
Case‐2	88/M	18 mm	LAmbre, 24 mm	During the procedure	Left atrium	Transeptal approach trapping	None

**FIGURE 1:**
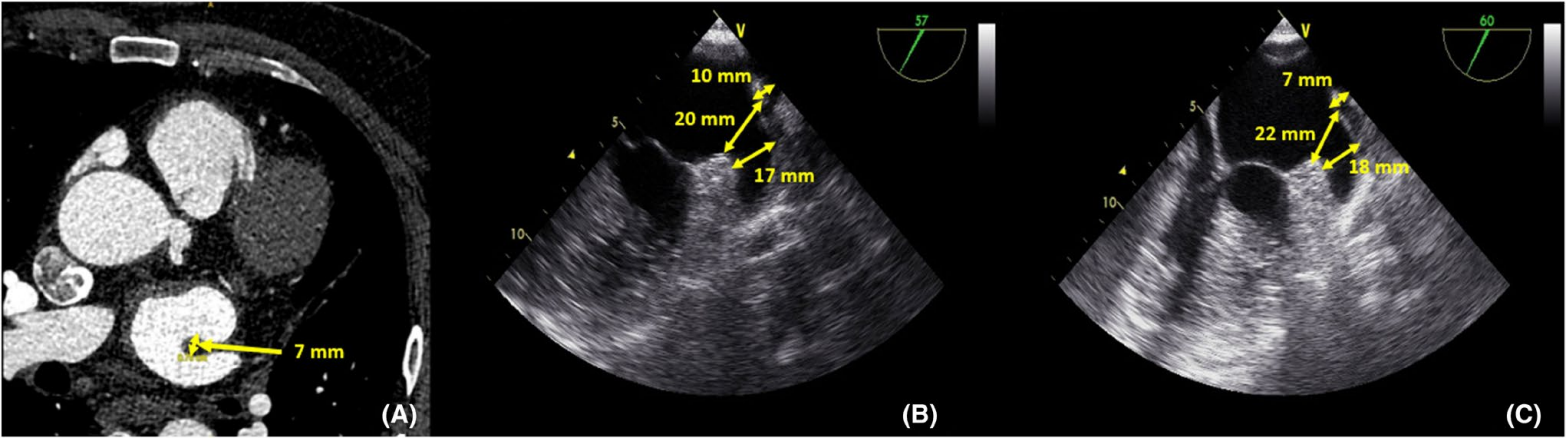
Serial imaging assessment demonstrating postcryoablation tissue changes and their impact on device sizing. (A) Preablation computed tomography showing baseline pulmonary ridge thickness of 7 mm (yellow arrow). (B) Transesophageal echocardiography at one‐month postcryoablation (at time of LAAC) demonstrating persistent tissue edema with increased pulmonary ridge thickness to 10 mm and landing zone measurement of 17 mm. Note the narrowed landing zone because of residual postablation swelling. (C) Follow‐up imaging at two months postablation showing resolution of tissue edema with pulmonary ridge thickness returning to baseline (7 mm) and landing zone expansion to 18 mm, illustrating how edema resolution contributed to relative device undersizing.

**FIGURE 2:**
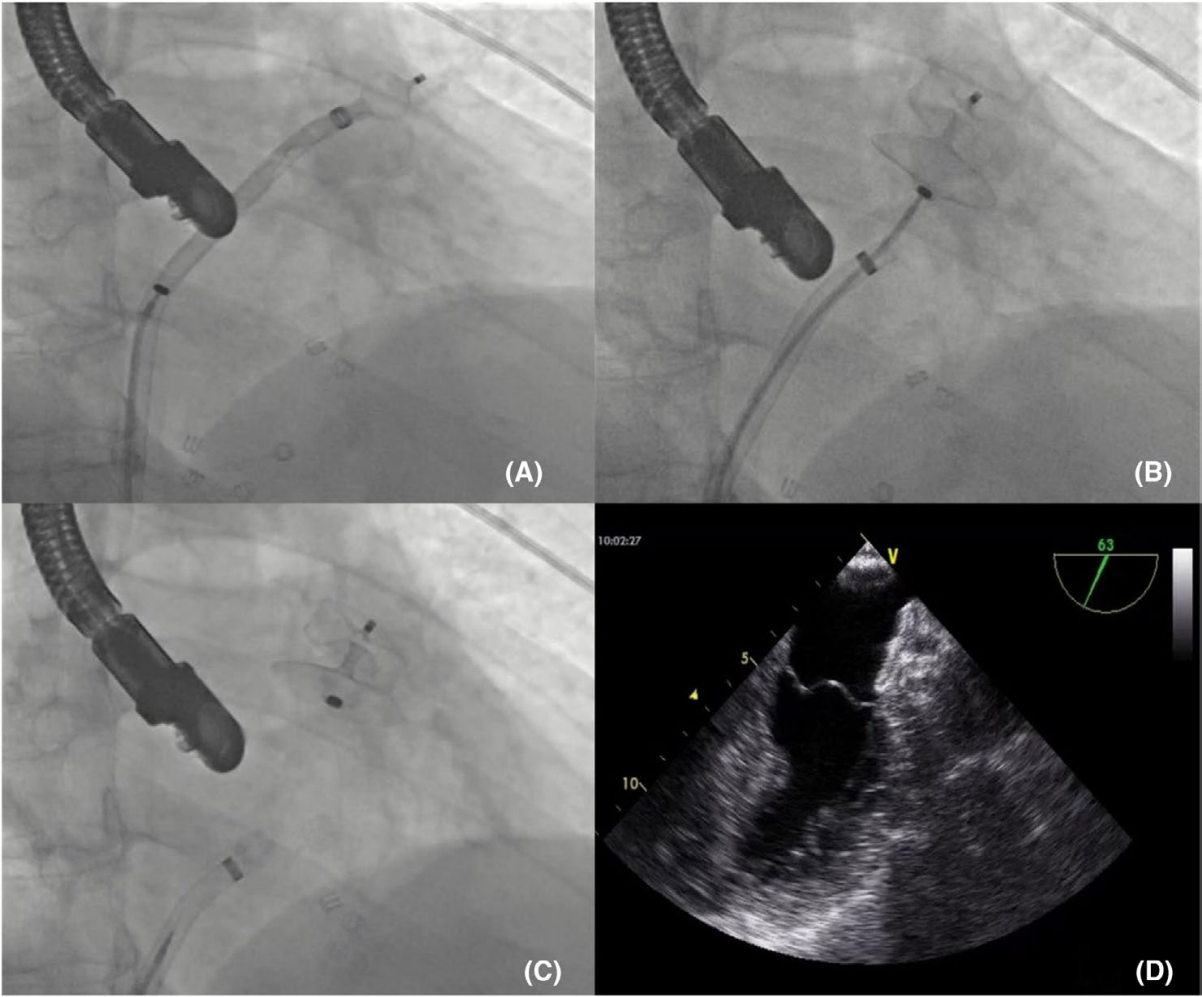
Left Atrial Appendage Closure (LAAC) with the Amulet™ Device in Case 1. (A) The delivery sheath demonstrates the ball‐shaped Amulet™ device. (B) Tug test confirming the stability of the device before release. (C) Final release of the device. (D) Intraoperative transesophageal echocardiography (TEE) image postdevice deployment showing proper positioning and sealing.

The patient underwent successful cryoballoon AF ablation, achieving isolation of all four pulmonary veins. The LAAC procedure was performed 1 month after the cryoballoon ablation. Before the LAAC procedure, the patient was given a rapid infusion of 500 cc of physiological saline. Preablation computed tomography showed a pulmonary ridge thickness of 7 mm (Figure 1A). At the time of the LAAC procedure (one‐month postcryoablation), transesophageal echocardiography revealed persistent tissue edema with increased pulmonary ridge thickness to 10 mm, representing a 43% increase from baseline (Figure 1B,C). The landing zone measured 17 mm at its narrowest diameter. A 20 mm (lobe diameter) AMPLATZER™ Amulet™ (Abbott, USA) device was used for LAAC (Figure 2). After the procedure, under transesophageal echocardiography (TEE) and fluoroscopy guidance, the disc's tire shape of the lobe, separation of the lobe from the disc, concavity of the disc, axis of the lobe, and the lobe is adequately around the circumflex artery seemed appropriate for device stability.^4^ At the same time, it was seen that the position and stability of the device were appropriate with light retraction maneuvers (Tension test) (Video S1). Following the procedure, the patient was discharged on a treatment regimen that included aspirin 100 mg, propafenone 2 × 150 mg, bisoprolol 5 mg, and pantoprazole 40 mg.

**FIGURE 3:**
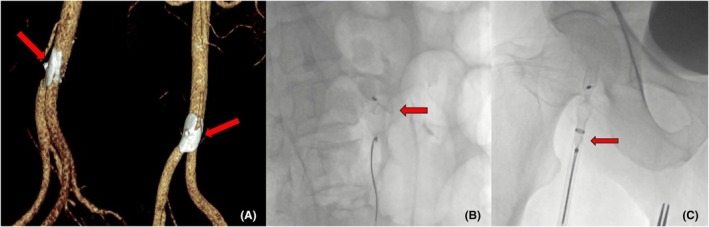
Detection of the Embolized Device via CT and retrieval using a snare catheter. (A) The device is observed in the terminal aorta. (B) The snare catheter is seen capturing the proximal portion of the device. (C) The device, secured by the snare, is being retracted into the 14F sheath.

One week after discharge, the patient presented to the emergency department with complaints of bilateral leg claudication. In control TTE, it was observed that the LAAC device was not in place, there was no pericardial fluid, and the device was not in the intracardiac chambers. A thoracic and abdominal computerized tomography (CT) angiography scan revealed that the LAAC device had migrated to the terminal aorta, and there was a type B aortic dissection flap starting from the distal left subclavian artery and extending to the distal thoracic aorta (Figure 3A). Percutaneous retrieval was planned, and a 14 French sheath was inserted through the right femoral artery. Despite initial challenges in capturing the device using 20 mm (Cook Medical Snare, USA) and 25 mm (Amplatz GooseNeck® Snare, Medtronic, USA) snares, it was manipulated retrogradely into the thoracic aorta and subsequently captured in the abdominal aorta. However, the device could not be inserted into the sheath and became lodged at the vascular access site. Using a clamp, the device was successfully withdrawn from the femoral artery (Figure 3B,C; Video S2). Control thoracic and abdominal CT angiography showed a stable aortic dissection flap starting from the distal left subclavian artery and extending to the distal thoracic aorta, along with focal dissections in the right external iliac and common femoral arteries. In the peripheral examination of the patient, bilateral lower extremity pulses were palpable.

No additional intervention was performed on the patient, and the remaining hospital stay was uneventful. The patient was admitted for a control visit 1 month later and had no complaints. In the control thoracic and abdominal CT angiography, it was seen that the dissection lines remained stable and did not progress. Redo‐LAAC was planned for the patient. The left atrial pressure was 12 mmHg before the device size selection. Follow‐up imaging at one‐month postablation demonstrated resolution of pulmonary ridge edema, with thickness returning to baseline at 7 mm. Simultaneously, the landing zone dimensions increased to 18 mm (Figure 1C), reflecting the resolution of compressive effects from tissue swelling. This 1 mm increase in landing zone diameter after edema resolution likely contributed to relative device undersizing and subsequent embolization. A 22 mm LAmbre™ (Lifetech Scientific) device was successfully deployed, and the patient was discharged without further complications. The patient had no complaints at the 1st and 6th month follow‐up visits. The device was monitored in situ during TTE controls. In control thoracic and abdominal CT angiography taken at the 6th month, it was seen that the LAAC device was in place, and the aortic dissection remained stable and did not progress.

### 2.2 Case 2

An 88‐year‐old male with a medical history, including diabetes mellitus, hypertension, persistent AF, nonischemic dilated cardiomyopathy, and complete atrioventricular block with a permanent pacemaker, was admitted to our clinic. The patient had a history of significant gastrointestinal (GI) bleeding while on apixaban at a dose of 2.5 mg twice daily. A 3/6 systolic murmur was noted on physical examination at the tricuspid area and rales at the lung bases. The patient weighed 65 kg, had a height of 167 cm, and had a body mass index (BMI) of 23.3 kg/m^2^ (Table 1). Laboratory tests revealed a serum creatinine level of 1.04 mg/dL, an estimated glomerular filtration rate greater than 60 mL/min/1.73 m^2^, and a hemoglobin level of 10.3 g/dL. The ECG demonstrated a ventricular‐paced rhythm with an underlying AF. TTE revealed an LVEF of 40%, moderate mitral regurgitation, severe tricuspid regurgitation, and a systolic pulmonary artery pressure of 60 mmHg. The patient had a history of massive GI bleeding under OAC treatment, so LAAC was planned for the patient.

**FIGURE 4:**
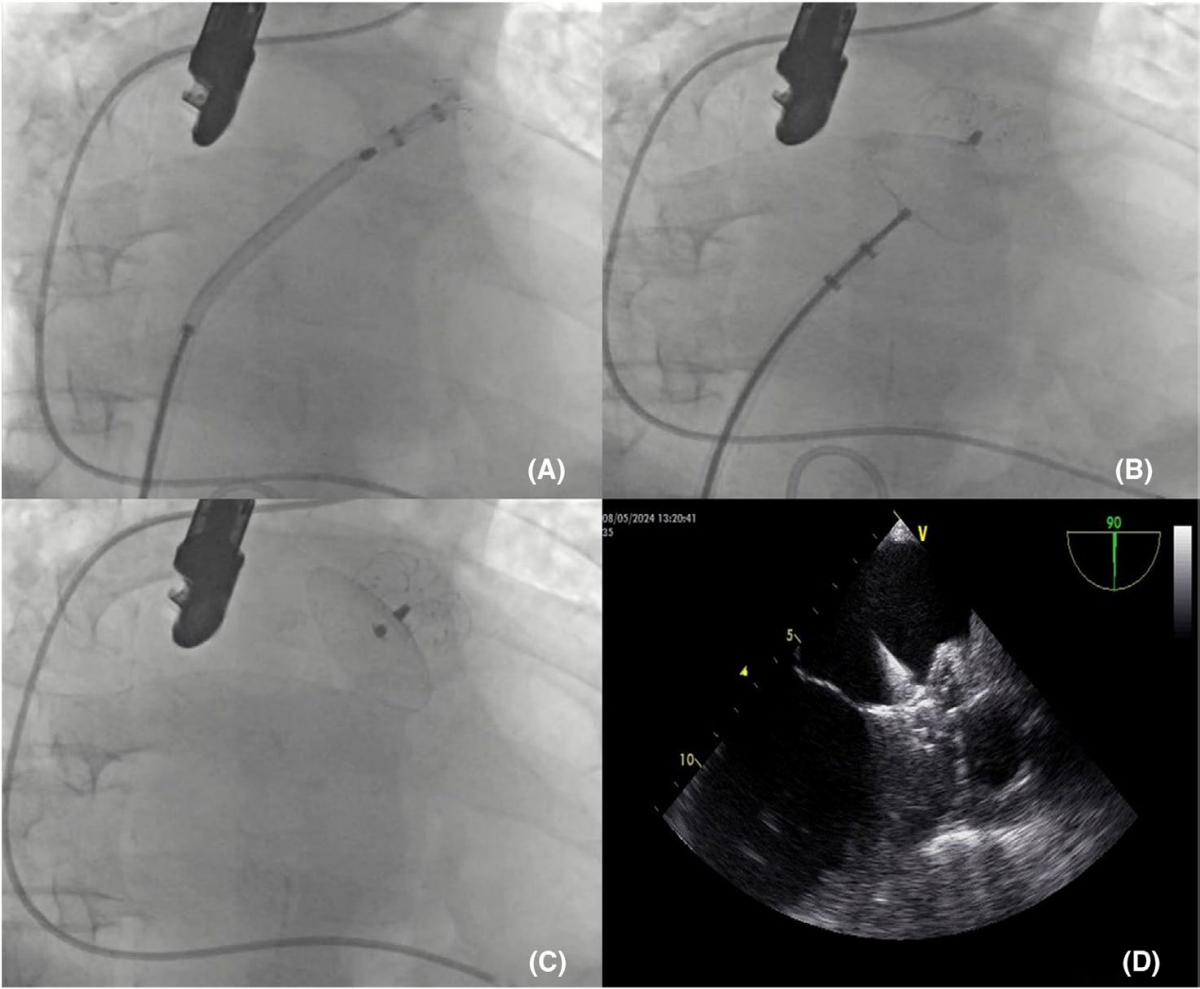
Left Atrial Appendage Closure (LAAC) with the LAmbre™ Device in Case 2. (A) The delivery sheath demonstrates the ball‐shaped LAmbre™ device. (B) Tug test confirming the stability of the device before release. (C) Final release of the device. (D) Intraoperative transesophageal echocardiography (TEE) image postdevice deployment showing proper positioning and sealing.

**FIGURE 5:**
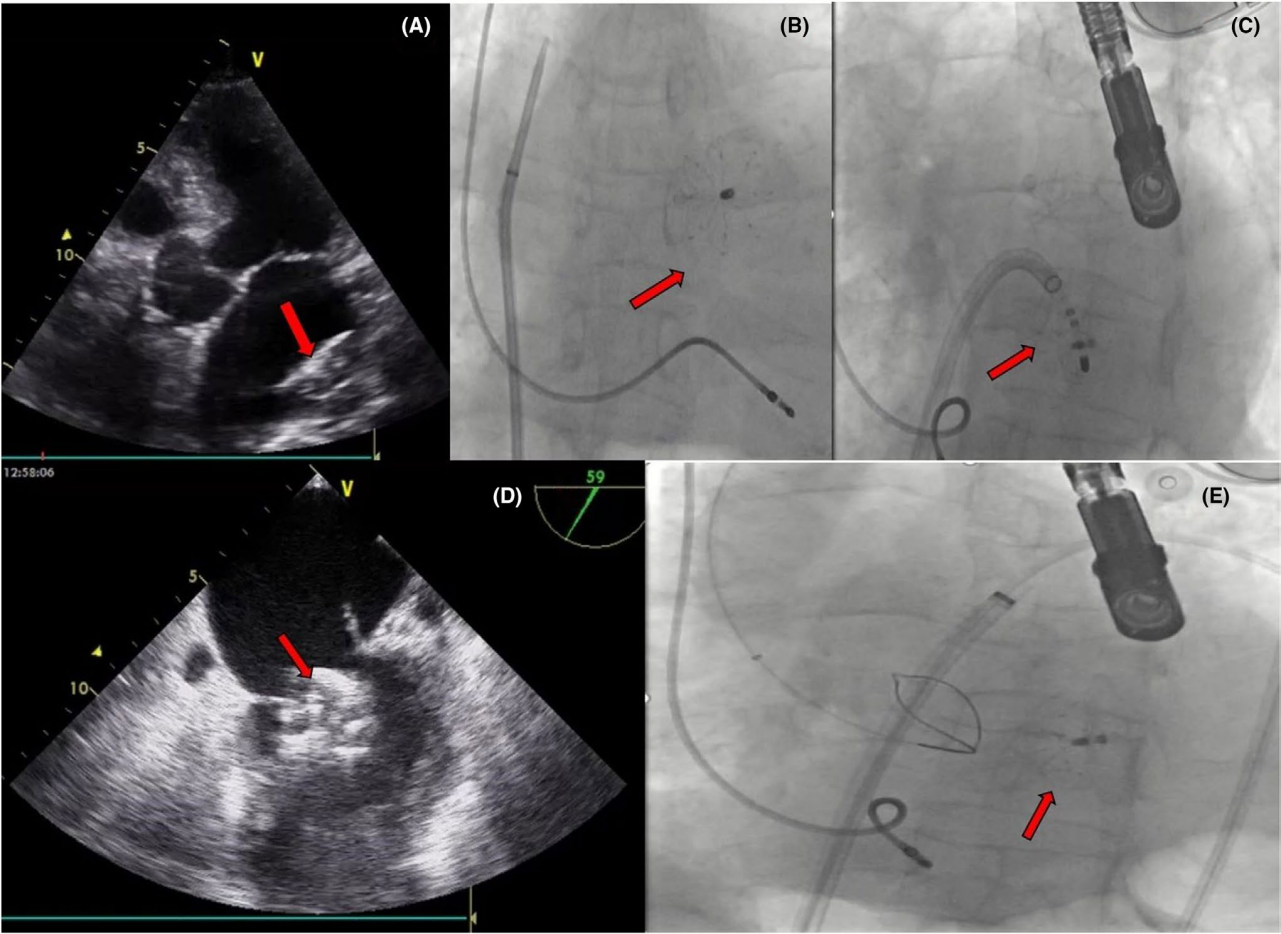
Echocardiographic and fluoroscopic images of the embolized device in Case 2 demonstrate various attempts to retrieve it. (A) T TTE image shows the device embolized in the left atrium. (B) Fluoroscopic image of the device freely floating within the left atrium. (C) An ablation catheter is being used to attempt to push the device into the left ventricle. (D) The device becomes trapped in the mitral valve, leading to acute severe mitral stenosis, as it is being pushed into the left ventricle in preparation for transaortic retrieval. (E) Unsuccessful attempt to capture the device transaortically using a snare catheter.

Preprocedural TEE identified a thrombus at the base of the LAA, along with dense spontaneous echo contrast. 500 cc of physiological saline was administered slowly before the procedure. The left atrial pressure was 16 mmHg before the device size selection. The landing zone on TEE was measured to be 18 mm at its widest point. A 24 mm LAmbre™ (Lifetech Scientific) device was successfully deployed (Figure 4). After deployment, a slight pullback (Tension) test confirmed the device was securely in place, and the color Doppler on TEE showed no residual flow in the LAA, indicating complete closure. The stability criteria of the device were met, and the device was left in place.^5^ However, in the immediate postprocedural period, before the patient fully awoke from anesthesia, TTE revealed that the device had migrated and was freely floating within the left atrium (Figure 5A).

**FIGURE 6:**
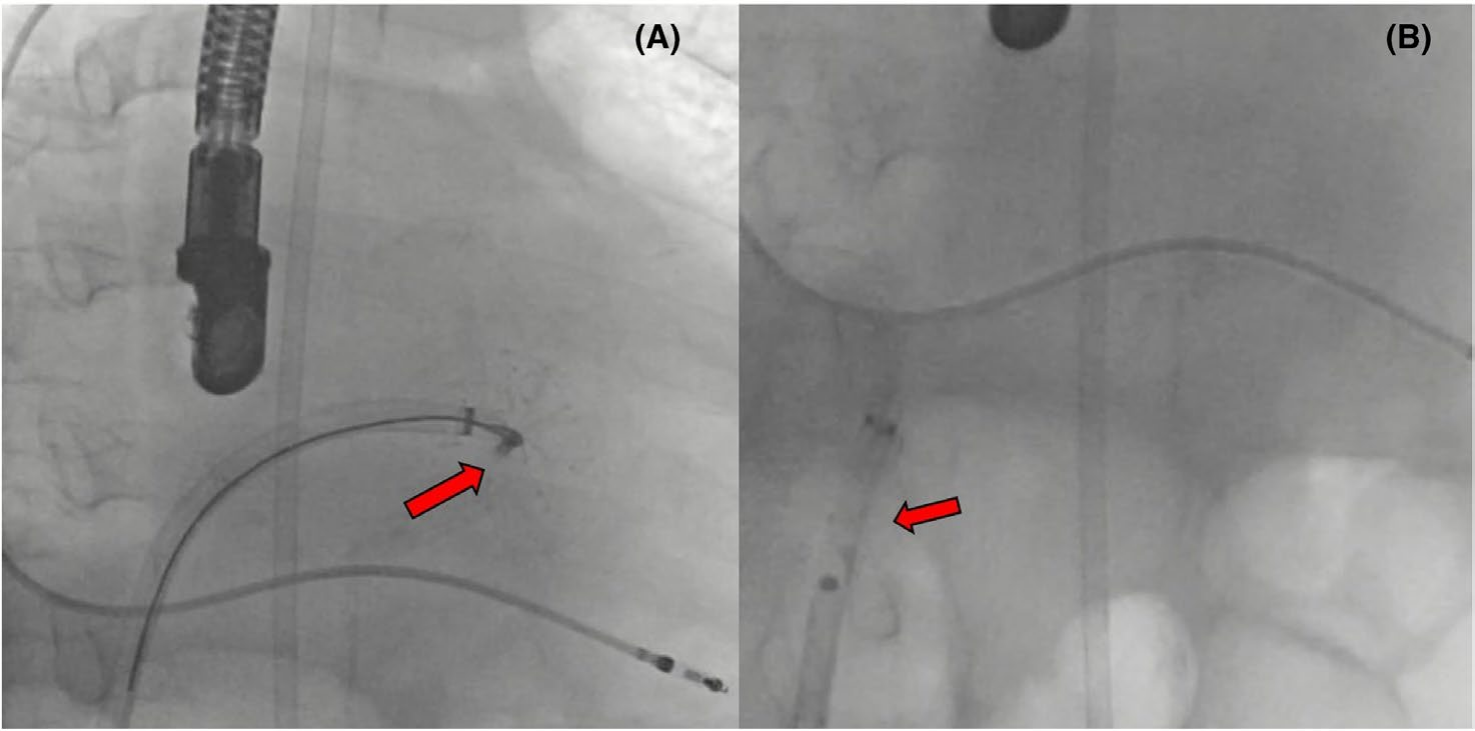
Transseptal retrieval of the embolized LAmbre™ device. (A) The device, trapped in the mitral valve, is captured by the snare catheter and retracted into the sheath. (B) The device, secured within the sheath, is successfully withdrawn along with the sheath.

An urgent attempt was made to retrieve the device. A repeat transseptal puncture was performed from a suitable alternative site, and the device was advanced into the left ventricle (LV) using the radiofrequency ablation Marinr catheter (Medtronic, Minneapolis, MN, USA) (Figure 5B,C). Efforts to capture the device using a transaortic approach with a 20 mm snare (Cook Medical Snare, USA) were complicated by the device becoming lodged in the mitral valve posterior apparatus (Figure 5D). Recognizing the potential for mitral valve injury, it was decided to abandon the transaortic approach. Instead, the device was retrieved using a snare and removed via a transseptal approach without further complications (Video S3). Using a transseptal puncture route, the device was grasped at the junction between the disc and the lobule over the mitral valve using the larger internal diameter FlexCath Advance Steerable Sheath (Medtronic, USA) and retrieved within the sheath and removed (Figure 6). A 22 mm LAmbre™ (Lifetech Scientific) device was deployed. The stability criteria of the device were met, and the device was left in place. There were no complications in the patient's follow‐up.

## 3 DISCUSSION

LAAC is a critical intervention in patients with AF who are at high risk for stroke but cannot tolerate long‐term OAC therapy. Several landmark trials, including the PROTECT AF and PREVAIL trials, have supported the effectiveness of LAAC.^6^ These studies demonstrated that LAAC is noninferior to OAC in preventing stroke and systemic embolization, particularly in high‐risk patients. However, the procedure is not without risks. Complications such as DE, pericardial effusion, and procedural stroke, while relatively rare, pose severe concerns for clinicians.

DE is a rare but potentially life‐threatening complication of LAAC, with an incidence rate reported between 0.6% and 1.5% in large‐scale studies.^7^, ^8^, ^9^ Risk factors for DE include improper device sizing, suboptimal device positioning, and structural abnormalities of the LAA. Previous expert consensus statements and guidelines emphasize the importance of meticulous procedural planning to prevent DE. The 2020 SCAI/HRS expert consensus on LAAC highlighted that proper device sizing and careful imaging using TEE and fluoroscopy are critical to minimizing the risk of embolization.^10^ A study by Reddy et al. highlighted that improper device sizing is a primary cause of DE, with larger devices being more prone to migration because of incomplete attachment to the LAA ostium.^3^ Furthermore, patients with extensive comorbidities, such as those with dilated cardiomyopathy or elevated pulmonary artery pressures, may be at increased risk for device instability and embolization.^10^ In the first case, the LAAC procedure was performed 1 month after cryoballoon ablation, allowing sufficient time for tissue healing and resolution of any procedure‐related edema. This staged approach was intended to minimize the risk of device embolization related to anatomical changes caused by ablation‐induced tissue swelling. Recent studies have demonstrated that ablation procedures, whether thermal or nonthermal, can cause significant acute tissue edema that may affect device sizing and stability. Tam et al. reported that even pulsed field ablation (PFA), despite being nonthermal, causes a mean 69.4% increase in pulmonary ridge thickness immediately following the procedure.^11^ This acute swelling can lead to inaccurate anatomical measurements during device sizing, potentially resulting in relative device undersizing once the edema resolves. In cryoballoon ablation, similar tissue changes occur. Ren et al. demonstrated that cryoballoon ablation increases left atrial ridge thickness by a mean of 2.6 mm.^12^ The acute inflammatory response and tissue edema following thermal ablation can persist for several weeks, making immediate device sizing challenging and potentially increasing the risk of device migration once the swelling subsides. The mechanism of postablation device embolization likely involves initial appropriate device sizing in the presence of tissue edema, followed by relative device undersizing as the edema resolves over time. This creates a mismatch between the device size and the actual landing zone dimensions, predisposing to device migration. Despite the one‐month interval in our case, the device embolization suggests that either residual tissue changes persisted or other factors such as device selection criteria or anatomical considerations contributed to the complication. The COMBINATION trial, which randomized patients undergoing combined radiofrequency ablation and LAAC into ablation‐first versus occlusion‐first strategies, found that peridevice leak rates were three times higher in the ablation‐first group, supporting the hypothesis that ablation‐induced tissue changes can adversely affect device performance.^13^ These findings underscore the importance of careful timing, meticulous device sizing, and consideration of staged procedures when combining ablation with LAAC.

The management of DE depends on the embolization timing and the migrated device's anatomical location. A systematic review by Eppinger et al. found that most DEs occur peri‐procedurally, with the majority detected immediately postprocedure or within the first few days of discharge.^9^ Management strategies often involve percutaneous retrieval using snares or forceps. Surgical retrieval is typically reserved for cases where percutaneous attempts fail or when the device has migrated to a location that makes percutaneous techniques impractical. In a study by Turagam et al., endoscopic grasping tools were highlighted as a practical option for retrieving embolized devices under challenging cases, minimizing the risk of damage to surrounding structures.^14^ The percutaneous DE retrieval success rate was high (immediate success rate of 68.8%) in a recent multicenter registry study. Operators typically used the transseptal approach for removing devices embolized in the LA, while the transarterial approach was preferred for devices embolized in the aorta. A surgical approach was generally favored for devices embolized in the LV because of the risk of damage to critical anatomical structures, such as the mitral valve.^9^

Moreover, a report by Ha et al. introduced a “double snare” technique as a successful method for device retrieval, particularly in cases where standard single‐loop snares are insufficient.^15^ Endomyocardial biopsy forceps have also been reported to be helpful in specific anatomical situations where snares cannot adequately grasp the device.^16^ More recently, Chugh et al. reported the first percutaneous use of the ONO retrieval system (ONOCOR LLC) to remove an embolized Amulet device.^17^ The ONO retrieval system has a large basket that expands to fill the vessel lumen, making capturing the target device coaxially with the introducer sheath easier. The ONO basket reduces the likelihood of accidental cardiac or vascular tissue injury during device manipulation and provides a mechanical advantage for compressing targeted objects to a smaller size.

In an experimental animal study, sixteen LAmbre LAA implants were released into the left atrium of healthy dogs to imitate DE. Devices embolized into the left atrium and aorta were successfully removed via a transseptal approach without complications. However, severe complications occurred in three cases where the device embolized into the LV. One case resulted in cardiogenic shock and death because of significant mitral valve damage. In contrast, the other two cases experienced severe aortic valve injury during percutaneous retrieval.^18^ In one reported case, an Amplatzer Cardiac Plug device embolized into the LVOT was successfully removed by aligning the hooks parallel to the aortic valve. However, a transient atrioventricular (AV) block occurred, likely because of compression of the AV node.^19^ In another case involving a patient with a bioprosthetic aortic valve, an embolized Amplatzer Cardiac Plug device was successfully removed from the LVOT using a transseptal approach, with a pigtail catheter placed in the LVOT to prevent further embolization into the ascending aorta.^20^

In our two presented cases, we observed DE in different anatomical locations, requiring distinct management approaches. These cases reflect the challenges discussed in the literature, particularly the role of prompt imaging and procedural planning in preventing embolization. While the literature supports using percutaneous techniques in managing DE, the varied success of these methods underlines the need for operator experience and flexibility in approach. Both cases presented in our study illustrate the rare but severe complication of DE following percutaneous LAAC. In our cases, the likely reason was that the device selection was either undersized because of tissue edema after catheter ablation or oversized. The complexity of managing this complication, especially in older patients with comorbidities, underscores the importance of immediate diagnosis and intervention.

In the first case, DE occurred shortly after the procedure, underscoring the critical importance of precise procedural planning, especially regarding device sizing and placement. Misalignment or incorrect sizing, as highlighted in multiple studies, can lead to immediate postprocedural complications such as device migration. In our case, the landing zone measurement was determined to be 17 mm based on preprocedural TEE. A 20 mm AMPLATZER™ Amulet™ device was selected following the manufacturer's guidelines, which recommend oversizing the device by 2–4 mm for stability. However, postablation tissue edema at the left atrial ridge may have temporarily altered the landing zone dimensions, potentially causing relative undersizing. This highlights the importance of reassessing anatomical measurements intraoperatively after cryoablation to optimize device selection and prevent embolization. Early detection of DE via imaging allowed for timely intervention, preventing major vascular or systemic complications. However, our patient presented 1 week after the LAAC procedure, and the embolized device caused a descending aorta dissection, and the device was stuck at the distal abdominal aorta. The successful use of a snare‐based retrieval method aligns with the literature, which supports percutaneous techniques as the preferred first‐line strategy for managing DE. This case also emphasizes the value of operator experience and the need for rapid intervention in such situations. A key aspect during device retrieval is ensuring the proper positioning of the device.

In some cases, manipulation may be required to adjust the device's angle for successful capture. In this instance, we manipulated the device retrogradely to achieve the appropriate angle. Although the device was captured using a snare, we could not retrieve it into the delivery sheath. As a result, we pulled the device back directly to the femoral artery. The hooked structure of the device likely contributed to the iliac and femoral artery dissection during this manipulation. While forceps could have been an alternative retrieval tool, they were unavailable then. Another important consideration is the need for aortic imaging when using a transaortic approach, as aortic dissection is a known potential complication. This case highlights the importance of thorough preparation and careful handling during device retrieval to minimize such risks.

The second case, involving an 88‐year‐old male with dilated cardiomyopathy and elevated pulmonary pressures, presented a significantly more complex challenge. In this patient, DE was likely aggravated by his underlying structural heart disease, which inherently increases the risk of device instability, as suggested by Saw et al.^10^ The combination of dilated cardiac chambers and increased pulmonary pressures likely contributed to the difficulty in maintaining the device position, underscoring the need for careful procedural planning and device selection in patients with similar profiles. The complexity of this retrieval, with the device becoming lodged in the mitral valve, required adaptability in the approach. Initially, a transaortic retrieval was attempted, but because of the high risk of valve damage, the approach was switched to a transseptal method, resulting in successful removal. This flexibility in procedural technique underscores the importance of tailoring strategies to the specific location of the embolized device and the patient's anatomical considerations. Similar cases of asymptomatic device migration through the mitral valve have been reported in the literature, further emphasizing the variability in device stability and embolization outcomes.^21^ This case highlights the need for personalized approaches and the ability to modify strategies during the procedure to ensure patient safety and successful outcomes.

While both cases were successfully managed regarding procedural outcomes, the broader context of the patient's overall health significantly influenced their long‐term prognoses. This serves as a reminder that the success of interventions like LAAC is not solely dependent on procedural technique but also on comprehensive perioperative management and close postprocedural monitoring to prevent complications unrelated to the procedure. Moreover, these cases illustrate that although percutaneous retrieval methods, such as using snares or specialized tools like biopsy forceps, are highly effective, the choice of technique should be guided by the anatomical location of the device and the patient's clinical stability. In our experience, both cases required different retrieval approaches based on the device's position and the risk of further complications, reinforcing the importance of having multiple tools and strategies at the disposal of the interventional team.

**FIGURE 7:**
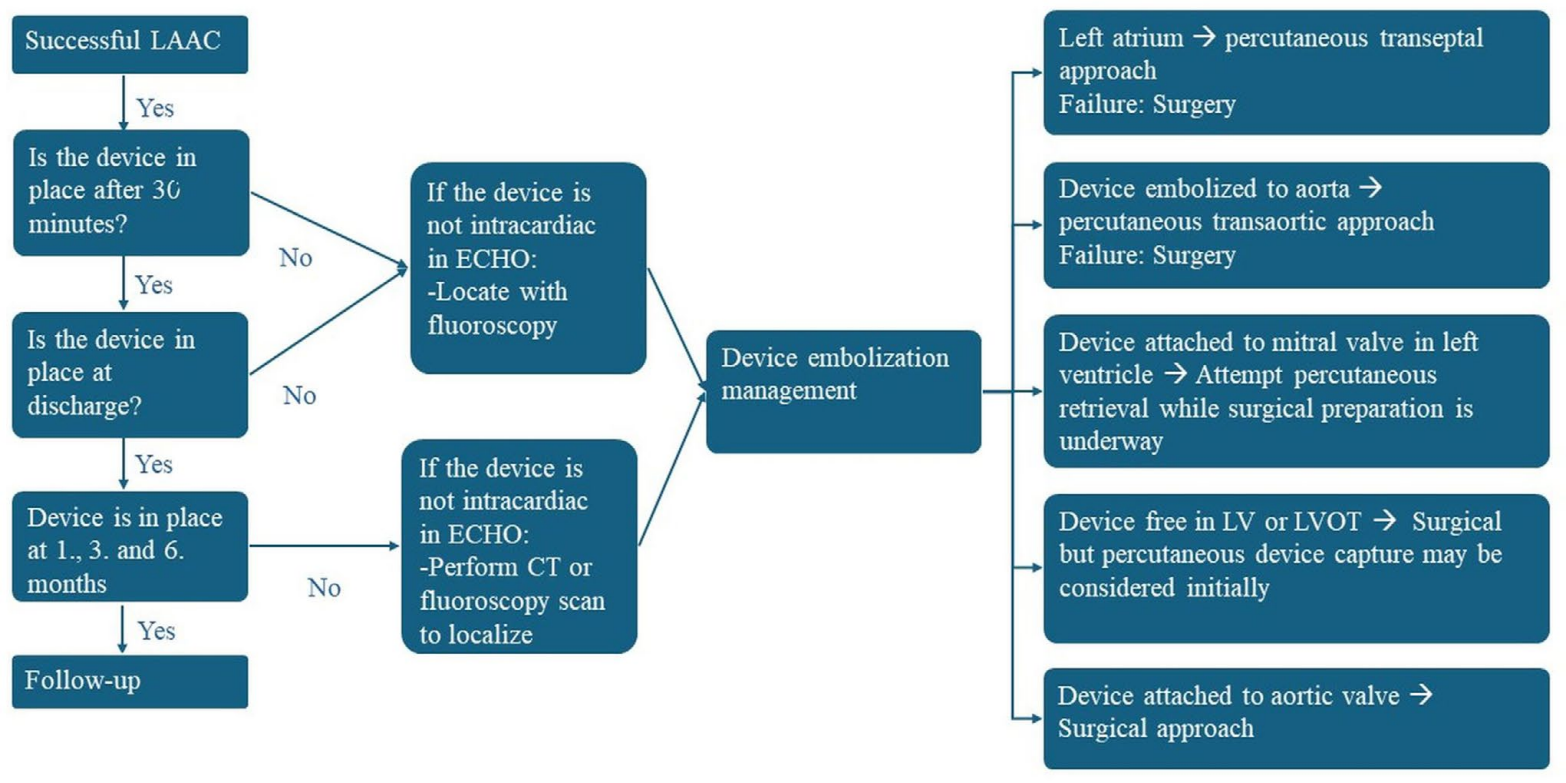
Our suggested algorithm for device embolization management after percutaneous left atrial appendage closure.

Looking ahead, it is clear that while the literature on LAAC continues to evolve, the need for a standardized algorithm for managing DE remains challenging. Studies such as those by Mansour et al. and Eppinger et al. emphasize the variability in DE management, from percutaneous methods to the occasional need for surgical intervention. These cases further demonstrate the importance of early detection, careful patient selection, and procedural planning to mitigate the risk of DE (Figure 7).

In conclusion, managing DE in LAAC requires a highly individualized approach, informed by the anatomical characteristics of the embolization and the patient's overall health status. Operator expertise, the availability of multiple retrieval tools, and the ability to adapt strategies based on real‐time clinical feedback are crucial for successful outcomes. Further research is needed to develop standardized protocols to guide clinicians in managing this challenging complication.

## FUNDING INFORMATION

The authors declared that this study has received no financial support.

## CONFLICT OF INTEREST STATEMENT

The authors declare they have no conflicts of interest.

## INFORMED CONSENT

Written informed consent was obtained from the patient (or the patient's legal representative) for the publication of this case report.

## Supporting information


**Video S1:** Fluoroscopic and echocardiographic images of the left atrial appendage closure (LAAC) device implantation in Case 1. The video shows the positioning and deployment of the device under real‐time fluoroscopy, followed by transesophageal echocardiography (TEE) imaging to confirm proper placement and stability within the left atrial appendage.

**Video S2:** Percutaneous retrieval of the embolized left atrial appendage closure (LAAC) device from the abdominal aorta in Case 1. The video details the step‐by‐step process of advancing a snare catheter through the femoral artery to capture the device in the aorta. Under fluoroscopic guidance, the device is successfully secured and retracted, showcasing precise maneuvering to avoid vascular injury during extraction.

**Video S3:** Transseptal retrieval of the embolized left atrial appendage closure (LAAC) device lodged in the mitral valve in Case 2. The video provides transesophageal echocardiography (TEE) and fluoroscopic views, clearly showing the device’s entrapment in the mitral valve. Under precise fluoroscopic guidance, the snare catheter is maneuvered transseptally to capture and safely retrieve the device while avoiding damage to the mitral valve apparatus.

## Data Availability

The data that support the findings of this study are available on request from the corresponding author.
